# Supplementary material for: Mode-multiplexing deep-strong light-matter coupling
Source: Nat Commun. 2024 Feb 28;15:1847. doi: 10.1038/s41467-024-46038-9 (PMC10901777; doi:10.1038/s41467-024-46038-9)
Supplement: Supplementary file 1 — Supplementary Information [file 41467_2024_46038_MOESM1_ESM.pdf]

Mode-multiplexing deep-strong light-matter coupling

Supplementary Information

Joshua Mornhinweg[1,2], Laura Katharina Diebel[1], Maike Halbhuber[1], Michael Prager[1], Josef Riepl[1], Tobias Inzenhofer[1], Dominique Bougeard[1], Rupert Huber[1,†], and Christoph Lange[2,†]

[1]*Department of Physics, University of Regensburg, 93040 Regensburg, Germany*

[2]*Department of Physics, TU Dortmund University, 44227 Dortmund, Germany*

Table of Contents

1. **Ultracompact metasurface design** 2
2. **Magnetoplasmon modes in two-dimensional electron gases** 5
3. **Multi-mode quantum model of deep-strong light-matter coupling** 10
4. **Subcycle time-domain quantum model** 15
5. **Parameter-free FEFD model** 18
6. **Scaling of the coupling strength** 21
7. **Additional data visualization** 22

1. Ultracompact metasurface design

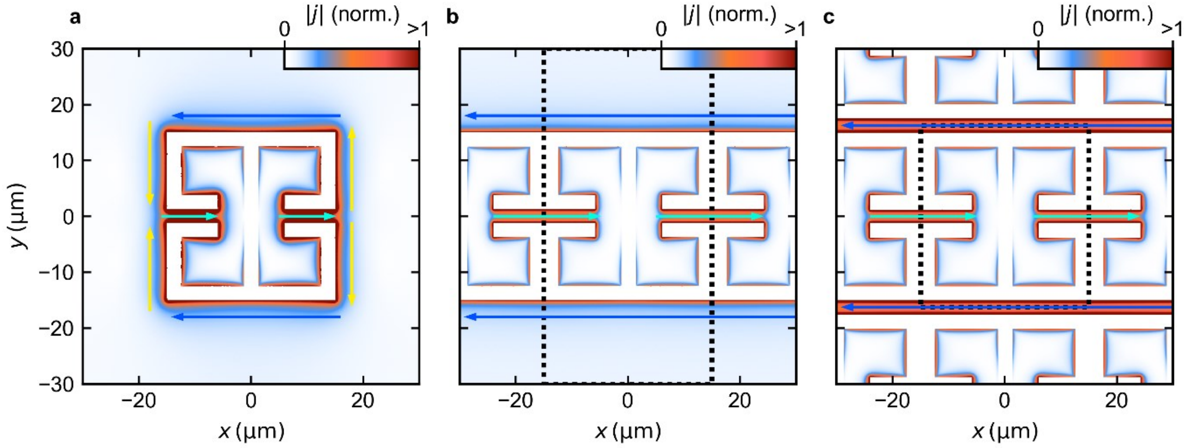

Fig. S1 | Ultracompact resonator design and current distributions. a, Resonator layout featuring a conventional, large unit cell. Arrows: Current flow of the fundamental optical mode. **b**, Resonator design compacted in x-direction, and current flow. The unit cell is indicated by the dashed rectangle. **c**, Resonator design, compacted in x and y-direction, and current flow.

The design of our gold metasurfaces aims at a maximally compact resonator geometry while providing spectrally well-separated optical modes with low linewidths, high near-field enhancement factors as well as low mode volumes. To this end, we have revisited some of the established design principles for plasmonic resonator structures. As detailed in the following, our approach enables excess spacing between adjacent resonators to be minimized or completely discarded, increasing the areal resonator density and thus the total oscillator strength of the metasurface by a factor of ~4 as compared to the original design.

The resonator structures are based on a square of 30 µm outer extension and twin current paths feeding a central capacitive gap of a width of 2.5 µm. The unit cell is twice as large as the outer dimensions of the resonator. In the frequency window of interest, the structure supports five optical modes with centre frequencies of 0.52, 1.95, 3.75, 4.6 and 6 THz (see main text, Table S1 and Fig. S2). The fundamental mode at 0.52 THz is excited by THz radiation linearly polarized in x direction, leading to an oscillating current flow along the paths indicated in Fig. S1a, whereby the current lags behind the driving field by a phase of π/2. As the field sweeps charges out of the right inner metal plate (right lime green arrow), symmetry dictates that a current identical in direction and magnitude drives charges into the

corresponding mirror plate on the left side of the structure (left lime green arrow). Following Kirchhoff's laws, the loop is closed by currents passing through the outer metal plane symmetrically, in y (yellow arrows) and x-direction (blue arrows). As a first step, we exploit the anti-symmetry of the y-oriented currents along the left and right edges of the outer metal plane. As the spacing of adjacent resonators in x-direction, s_x, is reduced, their near-field currents increasingly overlap. Owing to the opposite orientation of the currents marked by yellow arrows, they locally cancel each other. At the same time, the current flow into and out of the internal resonator area (lime green arrows) remains unaffected since the role of the currents marked in yellow is increasingly taken over by the current flows out of and into the internal resonator area of the nearest resonator neighbour. As a result, all relevant properties of the optical mode remain unaffected. Since this argument holds for any value of s_x, we chose s_x = 0, for which the metal area separating the resonators in x-direction vanishes completely. Here too, the currents exiting the inner metal plates of one resonator seamlessly continue to flow, now feeding the opposite metal plate of the adjacent resonator (Fig. S1b, lime green arrows) instead of the now absent y-polarized current paths (Fig. S1a, yellow arrows). Likewise, the x-polarized currents flowing along the outer perimeter of the structure are now connected between adjacent resonators (blue arrows) which also renders the y-polarized paths unnecessary.

We next consider a reduction of the spacing of the structures in y-direction, s_y. Since the outer currents of adjacent unit cells in y-direction carry the same phase, the cancellation exploited above cannot be applied here. However, since the resonance condition for the LC mode favours the shortest overall current paths, the currents are generally concentrated very close to the edges of the metallized layer (Fig. S1), from where they fall off within $\tilde{\rho}_y \approx 3$ µm in the direction normal to the edge, where $\tilde{\rho}_y$ is the characteristic decay length, in y-direction. As a consequence, s_y may be significantly reduced as long as the remaining metal stripline is of sufficient width to allow the x-polarized current to continue to flow. In the final structure, we chose s_y = 2.5 µm $\approx \tilde{\rho}_y$, leading to a reduction of the area of the unit cell by a factor close to 4. While the near-field distribution of this structure is virtually identical to that of the original one, the current distribution is markedly different. As Fig. S1 shows, the structure now exhibits two mainly x-polarized subcurrents which sweep charges between the adjacent inner metal

plates and along the striplines, respectively, and are offset by a phase of π. Finally, since the arguments of symmetry and current localization apply to all resonator modes equally, the spectrum of the compacted structure does not differ significantly from the spectrum of the separated structures across the entire spectral range, apart from coupling to surface plasmons [1] and the overall increase in transmission due to the unit cell reduction (Fig. S2).

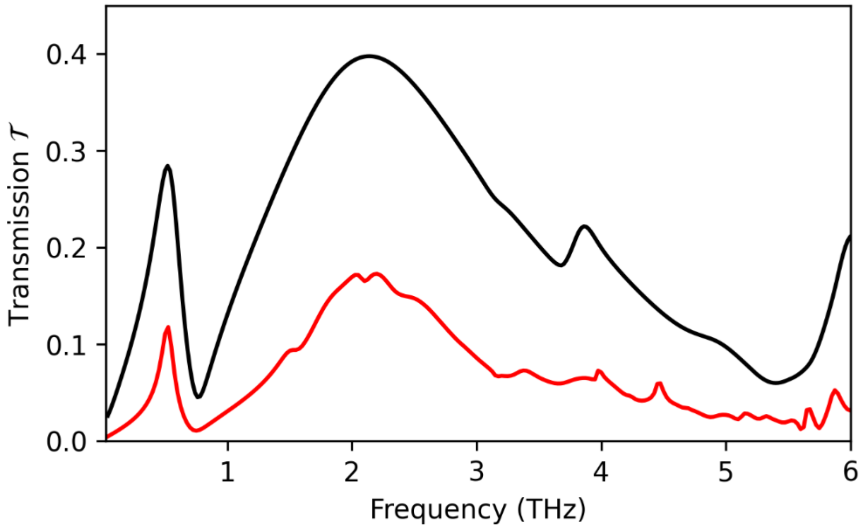

Figure S2 | Transmission for the bare resonators. Transmission spectrum for excitation in x-direction of the resonator array featuring conventional, large unit cell (60 µm x 60 µm, red curve) and of the compacted structure (black curve).

2. Magnetoplasmon modes in two-dimensional electron gases

In the two-dimensional electron gas (2DEG) hosted in our quantum wells (QWs), in absence of a magnetic field, plasma excitations obey the dispersion relation

$$\omega_{\mathrm{plasma}}(\mathbf{q}) = \sqrt{\frac{\rho_{\mathrm{2D}} e^2}{2m^* \epsilon_0 \epsilon_{\mathrm{eff}}(\mathbf{q})} |\mathbf{q}|}, \quad (1)$$

where $\mathbf{q}$ denotes the in-plane wavevector, ρ_{2D} is the two-dimensional (2D) electron density, m^* is the effective electron mass and $\epsilon_{\mathrm{eff}}(\mathbf{q})$ represents the effective dielectric constant for the electron gas [2,3]. Applying a static magnetic bias field oriented perpendicularly to the plane of the 2DEG introduces Landau quantization and the plasmon excitations hybridize with the cyclotron resonance, ω_{c}, resulting in the formation of magnetoplasmons (MPs) [4] with a frequency of

$$\omega_{\mathrm{MP}} = \sqrt{\omega_{\mathrm{c}}^2 + \omega_{\mathrm{plasma}}^2}. \quad (2)$$

The effective dielectric constant $\epsilon_{\mathrm{eff}}(\mathbf{q})$ for the MPs is set by the dielectric environment of the electron gas in the layers enclosing the 2DEG. In our structures, we consider the dielectric constant of the GaAs substrate below the QWs, $\epsilon_{\mathrm{sub}} = 12.9$, the capping layer of a thickness of d and dielectric constant $\epsilon_{\mathrm{barrier}}$ on top of the QWs, as well as the dielectric function of the top interface to either vacuum, or the gold parts of the resonator structure. The latter two possibilities have been analysed previously [5,3], resulting in corresponding effective dielectric functions, $\epsilon_{\mathrm{ungated}}(\mathbf{q})$ for the vacuum interface, and $\epsilon_{\mathrm{gated}}(\mathbf{q})$ for a top-metallized 2DEG:

$$\epsilon_{\mathrm{ungated}}(\mathbf{q}) = \frac{\epsilon_{\mathrm{sub}}}{2} + \frac{\epsilon_{\mathrm{barrier}}}{2} \times \frac{1 + \epsilon_{\mathrm{barrier}} \tanh(|\mathbf{q}|d)}{\epsilon_{\mathrm{barrier}} + \tanh(|\mathbf{q}|d)} \quad (3)$$

$$\epsilon_{\mathrm{gated}}(\mathbf{q}) = \frac{\epsilon_{\mathrm{sub}} + \epsilon_{\mathrm{barrier}} \coth(|\mathbf{q}|d)}{2}. \quad (4)$$

Depending on the situation, either of the two functions assumes the role of $\epsilon_{\mathrm{eff}}(\mathbf{q})$ in Eq. 1. For more complex, laterally structured samples such as planar metal resonators or gratings, an averaged, effective dielectric function can be constructed, proportionally factoring in the two situations according to a factor δ describing the relative metal coverage of the surface:

$$\epsilon_{\mathrm{eff,mix}}(\mathbf{q}) = \delta\epsilon_{\mathrm{gated}}(\mathbf{q}) + (1-\delta)\epsilon_{\mathrm{ungated}}(\mathbf{q}). \tag{5}$$

Moreover, the multi-QW stack consists of several layers with varying dielectric properties, requiring additional averaging of the effective dielectric function along the growth direction. As was shown previously, the response of the densely packed QWs is well approximated by an effective-medium approach [6]. In addition, as the QW stack exhibits a finite extension in growth direction, the charge carriers in the QWs are not an ideal 2D electron gas. More precisely, with increasing QW thickness, their plasma frequency approaches the 3D plasma frequency as an asymptotical upper limit for large wave vectors, whereas the dispersion of an ideal 2D plasma has no such upper bound. To account for this effect, we must include a correction which depends on the QW stack thickness t. The effective dielectric function ϵ_{eff} then is given by [7]:

$$\epsilon_{\mathrm{eff}} = \epsilon_{\mathrm{eff,mix}} + \frac{\epsilon_{\mathrm{sub}}\,|\mathbf{q}|t}{2}. \tag{6}$$

This description allows for a MP mode dispersion approaching the 3D plasma frequency asymptotically for $|\mathbf{q}| \to \infty$. However, plasmon and magnetoplasmon excitations are limited in frequency and wave vector by Landau damping which becomes effective when single-particle excitations become relevant [8,4]. An upper bound for the frequency of these single-particle excitations is given by $\nu < v_{\mathrm{F}} q_x$, with the Fermi velocity $v_{\mathrm{F}} = \frac{\hbar\sqrt{2\pi\rho_{2\mathrm{D}}}}{m^*}$.

The periodicity of our metasurfaces implies a discretization of the plasmon wave vectors that can be excited by the cavity modes. The corresponding condition for the in-plane wave vectors $\mathbf{q}_x$ is

$$|\mathbf{q}_x(\alpha)| = \frac{2\pi}{L_x}\alpha. \tag{7}$$

Here, L_x denotes the unit cell size of the structure in x-direction, and $\alpha \in \mathbb{Z}$ is the plasmon mode index. Linear combinations of plasmon waves with wave vectors $-\mathbf{q}_x$ and $\mathbf{q}_x$ form bright and dark standing waves $\Psi_{\mathrm{b}} \propto \exp(-i\mathbf{q}_x x) + \exp(i\mathbf{q}_x x)$ and $\Psi_{\mathrm{d}} \propto \exp(-i\mathbf{q}_x x) - \exp(i\mathbf{q}_x x)$, respectively. Since only the bright modes couple to the cavity modes, the dark modes are not further considered. We verify this approach for our complex resonator geometry by calculating the 2D Fourier transform of the x-polarized electric near-field component $\mathcal{E}_x$ of the first two cavity modes (Fig. S3a,b) along the x-direction. The

field $\mathcal{E}_x$ was calculated by the finite-element (FEM) method (see chapter 4) [6] and evaluated within xy-oriented planes that lie within the QW stack, whereby the doping concentration of the QWs was set to zero to obtain the modes of the empty cavity. This choice represents the field component most relevant for light-matter coupling, whereas we neglect the $\mathcal{E}_y$-polarized components since they are significantly weaker in most areas of the structure. The resulting amplitudes, integrated within the respective plane, are shown in Fig. S3c,d as a function of the wave vector and the depth below the metasurface. Owing to the spectrum of single-particle excitations (see previous paragraph) and the fact that the field amplitude decays for larger wave vectors, in particular within planes more distant to the metasurface, we limit the magnetoplasmon mode index α to a maximum of $|\alpha| \leq \alpha_{\mathrm{c}} = 10$. The data allows us to model the coupling of the light field to each QW separately and to account for the relative amplitude of each magnetoplasmon resonance. The same analysis is also performed for the higher resonator mode at 1.95 THz. The resulting plasmon dispersions, the wavevectors fulfilling the discretisation and the cut-off wavevector is shown in Fig. S4, whereas the relative coupling strengths for the plasmon modes in shown in the main manuscript Fig. 3a.

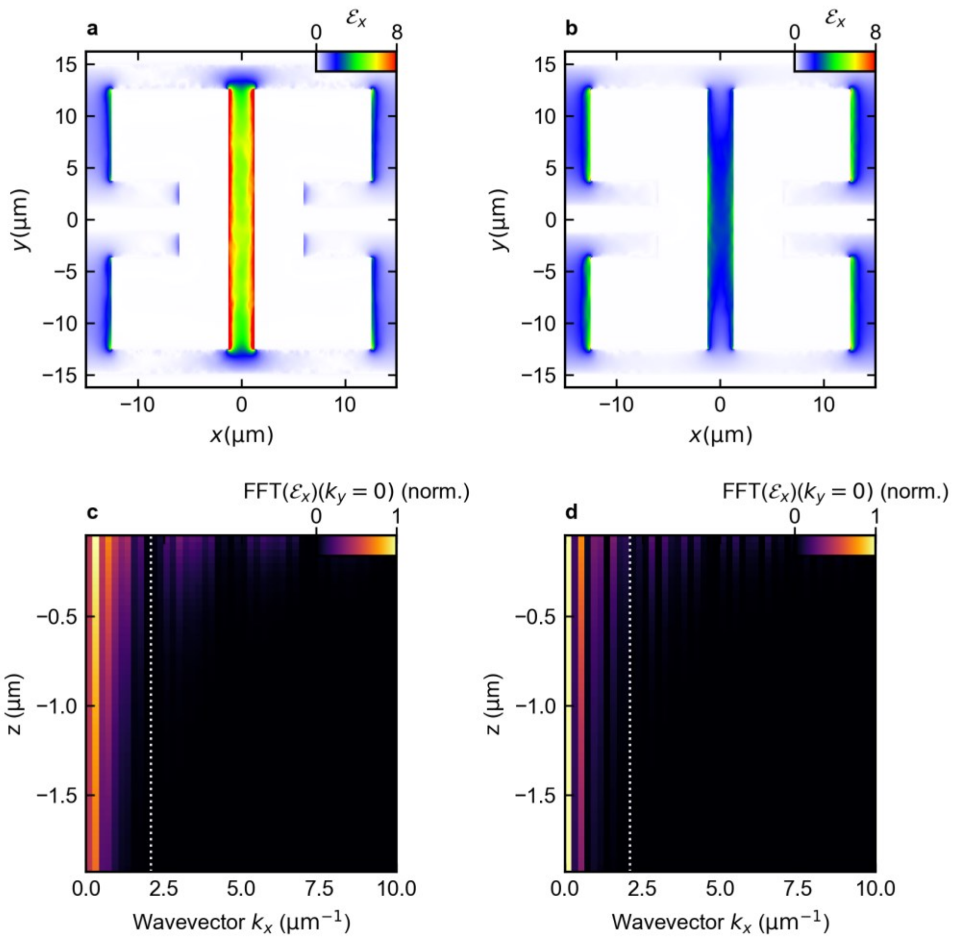

Figure S3 | Analysis of magneto-plasmon formation. Electric field component $\mathcal{E}_x$ in the quantum well plane 50 nm below the resonator structure, for **a**, the LC mode (*j* = 1) at 0.52 THz and **b**, the higher-order mode (*j* = 2) at 1.95 THz. **c**, Amplitude components of the Fourier-transformed data of panel **a** as a function of the wave vector component k_x, for $k_y = 0$, and the depth of the plane below the metasurface, z. **d**, Equivalent amplitude components for the data of the higher-order mode in panel **b**.

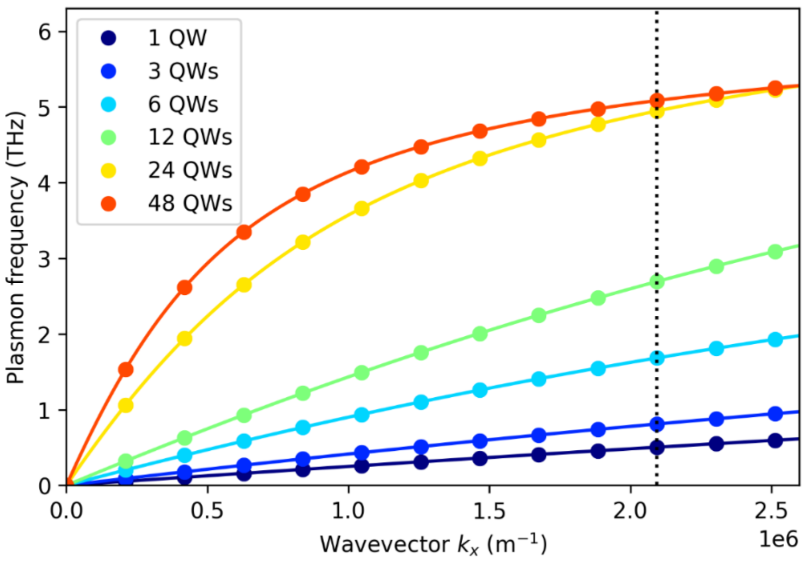

Figure S4 | Plasmon dispersion for all samples. The dots mark the plasmon frequencies which fulfil the diffraction condition. The black dashed line indicates the cut-off wavevector up to which plasmon modes were considered in the quantum model.

3. Multi-mode quantum model of deep-strong light-matter coupling

We describe the multi-mode light-matter coupling of our structures by a specifically developed mean-field theory treating light-matter interaction in the deep-strong coupling regime, including anti-resonant terms. We start with the Hamiltonian

$$\hat{H} = \hat{H}_{\mathrm{cav}} + \hat{H}_{\mathrm{e}} + \hat{H}_{\mathrm{int}} + \hat{H}_{\mathrm{dia}} + \hat{H}_{\mathrm{ext}}. \tag{8}$$

The cavity contribution is described by

$$\hat{H}_{\mathrm{cav}} = \hbar\omega_{\mathrm{cav}}\hat{a}^{\dagger}\hat{a}, \tag{9}$$

where ω_{cav} is the frequency of the cavity mode, and $\hat{a}$ is the corresponding bosonic annihilation operator. In a first limit of low doping density ρ, the frequencies of plasma excitations are much lower than the linewidth of the cyclotron resonance, such that the entire spectrum of excitations approximately coincides with the CR frequency. We implement this situation in the bosonic limit by

$$\hat{H}_{\mathrm{e}} = \hbar\omega_{\mathrm{c}}\hat{b}^{\dagger}\hat{b}. \tag{10}$$

Here, $\omega_{\mathrm{c}} = 2\pi\nu_c$ denotes the cyclotron frequency and $\hat{b}$ is the annihilation operator of the Landau excitations. Light-matter coupling including anti-resonant interaction terms is introduced by

$$\hat{H}_{\mathrm{int}} = \hbar\Omega_{\mathrm{R}}\left(\hat{a}^{\dagger} + \hat{a}\right)\left(\hat{b}^{\dagger} + \hat{b}\right), \tag{11}$$

where Ω_{R} is the vacuum Rabi frequency of the cavity mode coupling to the cyclotron resonance. The blue-shift of the cavity modes by diamagnetic interactions is accounted for by

$$\hat{H}_{\mathrm{dia}} = \hbar D\left(\hat{a} + \hat{a}^{\dagger}\right)^2, \tag{12}$$

where $D = \frac{(\Omega_{\mathrm{R}})^2}{\omega_{\mathrm{c}}}$. Coupling of the cavity modes to the THz far-field is contained in $\hat{H}_{\mathrm{ext}}$.

When the doping density is raised to a significant level, the plasmon frequencies increase such that $\omega_{\mathrm{plasma}}(\mathbf{q}(\alpha_{\mathrm{c}}))$ eventually exceeds the linewidth of the cyclotron resonance. As a consequence, the magnetoplasmon modes become the relevant matter excitations in the light-matter coupling Hamiltonian. At the same time, the multiple optical resonances of the resonator structure over several octaves of coupled modes have to be considered. We account for this situation by extending $\hat{H}_{\mathrm{cav}}$ to all cavity modes j with frequencies ω_j, while $\hat{H}_{\mathrm{e}}$ is modified to include all relevant MP resonances up to

the cut-off MP index, whereby the magnetoplasmon frequencies are $\omega_{\mathrm{MP}}(\alpha) = \sqrt{\omega_{\mathrm{c}}^2 + \omega_{\mathrm{plasma}}^2(\mathbf{q}(\alpha))}$.

Each cavity mode j is coupled to all matter modes simultaneously with an individual Rabi frequency $\Omega_{\mathrm{R},j,\alpha}$. The extended multi-mode Hamiltonian then reads:

$$\begin{aligned}\hat{\mathcal{H}} = \sum_j \hbar\omega_j \hat{a}_j^\dagger \hat{a}_j \; + \; \sum_\alpha \hbar\omega_{\mathrm{MP}}(\alpha)\hat{b}_\alpha^\dagger \hat{b}_\alpha + \; \sum_{\alpha,j} \hbar\Omega_{\mathrm{R},j,\alpha}\left(\hat{a}_j^\dagger + \hat{a}_j\right)\left(\hat{b}_\alpha^\dagger + \hat{b}_\alpha\right) \\ + \sum_{\alpha,j} \frac{\hbar\Omega_{\mathrm{R},j,\alpha}^2}{\omega_{\mathrm{MP}}(\alpha)}\left(\hat{a}_j^\dagger + \hat{a}_j\right)^2 + \hat{\mathcal{H}}_{\mathrm{ext}}.\end{aligned} \tag{13}$$

The individual coupling strengths of the MP modes, $\Omega_{\mathrm{R},j,\alpha}$, are determined by the amplitudes of the associated wave vectors, as obtained by a Fourier transform of the near field of the resonator mode (see previous section). The set of all $\Omega_{\mathrm{R},j,\alpha}$ is then scaled by a single common factor to match the experimental spectra.

We perform a Bogoliubov transformation in order to determine the frequencies of the coupled modes as well as their light-matter composition, which is given by the eigenvalues and eigenvectors of the transform matrix. The resulting normal-mode polariton operators, $\left\{\hat{p}_{\beta,j}, \hat{p}_{\beta,j}^\dagger\right\}$, are given by $\hat{p}_{\beta,j} = w_{\beta,j}\hat{a}_j + \sum_\alpha x_{\beta,\alpha}\hat{b}_\alpha + y_{\beta,j}\hat{a}_j^\dagger + \sum_\alpha z_{\beta,\alpha}\, \hat{b}_\alpha{}^\dagger$ and generally contain contributions from all magnetoplasmon modes. The Hopfield coefficients $(w_{\beta,j}, x_{\beta,\alpha}, y_{\beta,j}, z_{\beta,\alpha})$ represent the polariton fractions corresponding to the bare cavity and matter modes. The absolute values of the Hopfield coefficients are shown in Fig. S5 for the sample with 48 QWs, for a cyclotron frequency of $\nu_{\mathrm{c}} = 0.52$ THz. As discussed in the main text, each wave vector pair $(-\mathbf{q}_x, \mathbf{q}_x)$ enables two linear superpositions associated with a dark and a bright magnetoplasmon mode, respectively. For clarity, we enumerated the eigenmodes of the Hamiltonian as follows: dark magnetoplasmon modes which do not couple to the cavity modes have a polariton index $\beta < 0$. The lower polariton mode is attributed to $\beta = 0$. Finally, the bright magnetoplasmon modes which do couple to the cavity field lead to the light-matter hybridized upper polariton modes with indices $\beta > 0$. Following the procedure of Ref. [9], we subsequently calculate the expectation value of the vacuum photon population $\langle N\rangle = \langle G|\hat{a}_j{}^\dagger \hat{a}_j|G\rangle$ for

each cavity mode j, whereby $|G\rangle$ denotes the ground state of the coupled system. For our structures, the vacuum photon population $\langle N\rangle$ not only reaches values of up to one entire photon, but the vacuum ground state moreover features exotic properties such as a non-classical Fock state occupation distribution. More precisely, the occupation probability does not diminish monotonically as a function of the photon number but instead shows a higher occupation probability for the excited state $|2\rangle$ as compared to the state $|1\rangle$ (Fig. S6a). In addition, the ground state exhibits strong squeezing as evidenced by the Wigner function for the photonic state (Fig. S6b-d).

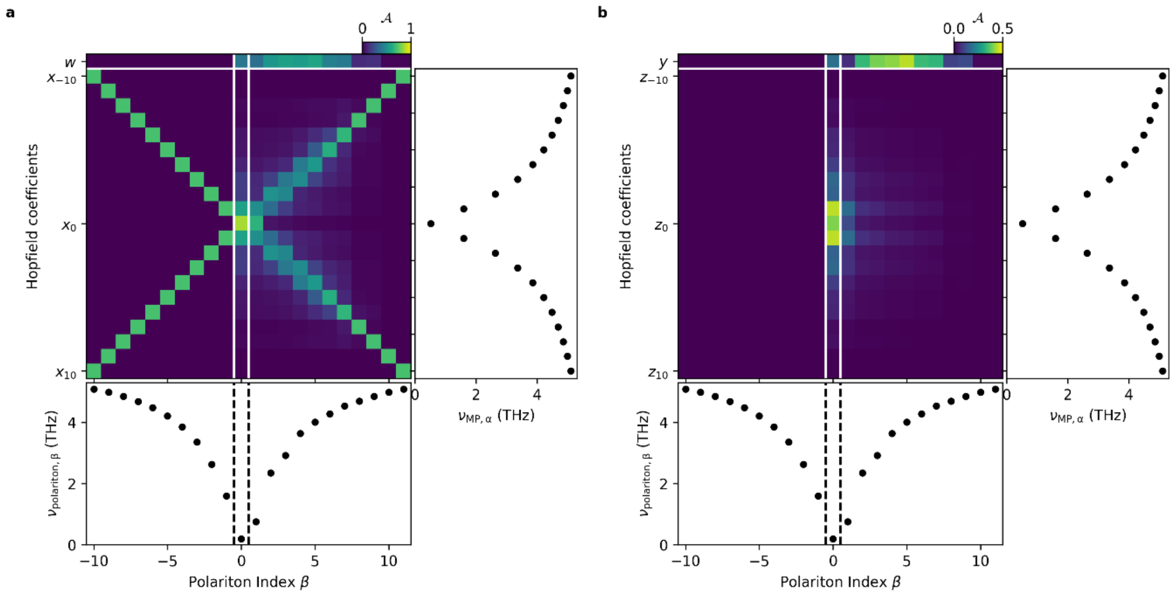

Figure S5 | Hopfield coefficients for the 48-QW structure. **a**, Plot of the absolute values of the complex-valued Hopfield coefficients for the lowest cavity mode, $j = 1$, and a cyclotron frequency of $\nu_c = 0.52$ THz. The right and bottom panels show the frequencies of the uncoupled magnetoplasmon and the coupled polariton modes, respectively. w: light mode, x_{-10} to x_{10}: plasmon modes. Polariton index $\beta < 0$: uncoupled magnetoplasmon modes, $\beta = 0$: lower polariton, $\beta > 0$: upper polaritons. **b**, Absolute values of the anti-resonant Hopfield coefficients. y: light mode, z_{-10} to z_{10}: plasmon modes.

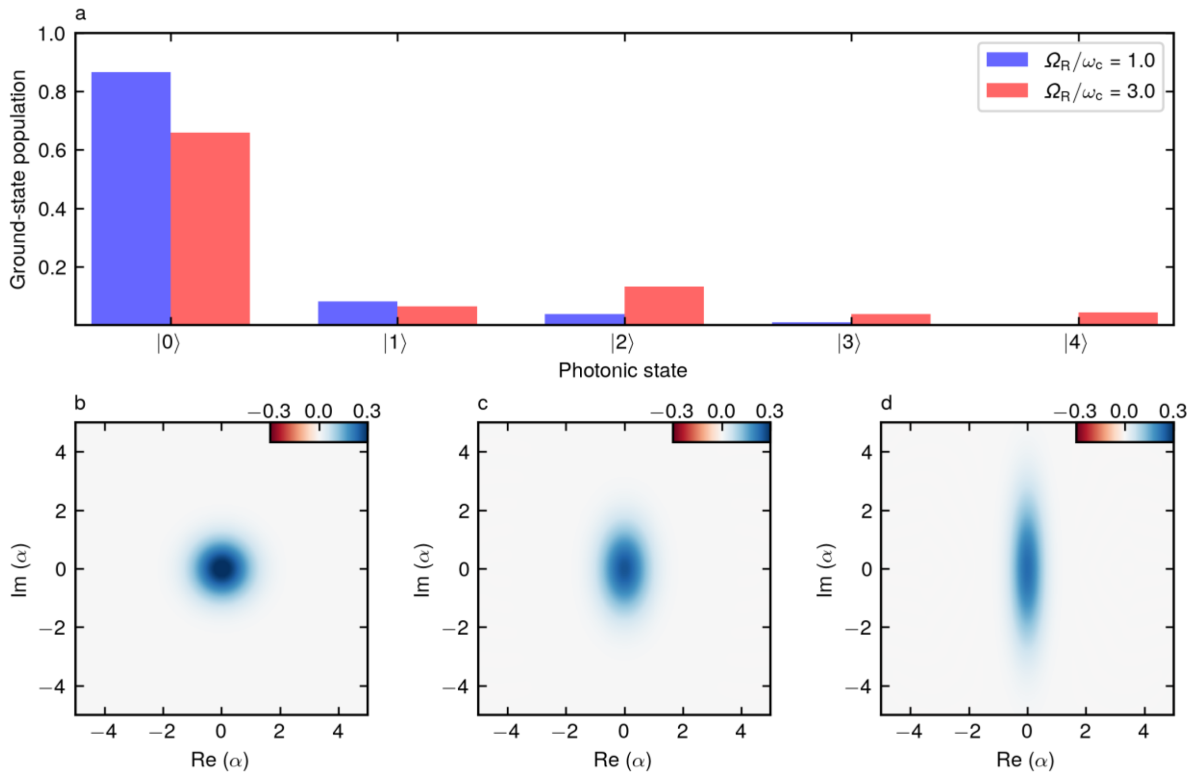

Figure S6 | Virtual photon occupation and squeezing of the vacuum ground state. The calculation considers a single cavity mode coupled to a single matter excitation, for clarity. **a**, Virtual photon population with a coupling strength of $\Omega_R/\omega_c = 1$ (blue bars) and $\Omega_R/\omega_c = 3$ (red bars). **b-d**, Wigner function of the photonic state for $\Omega_R/\omega_c = 0$ (**b**), $\Omega_R/\omega_c = 1$ (**c**) and $\Omega_R/\omega_c = 3$ (**d**).

Moreover, we investigate the virtual photon population $\langle N_1 \rangle$ as a function of the detuning of light and matter modes. To this end, we first calculate $\langle N_1 \rangle$ for a single cavity mode with a frequency of $\nu_{j=1}$ = 0.52 THz which is coupled to a single electronic excitation with a variable frequency ν_c, whereby we assume a coupling strength of $\Omega_{R,1}/\omega_1 = 2.83$ to approximate the situation of the structure with 48 QWs (Fig. S7a, dotted grey curve). Remarkably, even for a significant detuning of $\nu_c = 10 \times \nu_{j=1}$, the vacuum photon population remains above 50% of its maximum value found for $\nu_c = \nu_{j=1}$. The situation is even more favourable for the actual multi-mode setting of our structure, where tuning of the cyclotron resonance ν_c to values $\nu_c > \nu_{j=1}$ increases the detuning for some modes, while the detuning for others is reduced. As a result, the vacuum photon population (Fig. S7a, solid black curve) lies even slightly above the one for the single-mode scenario.

The situation changes considerably for moderate coupling strengths. We perform the calculation for a single pair of light and matter modes for $\Omega_{R,1}/\omega_1 = 0.1$. Here, a much stronger dependence of $\langle N_1 \rangle$ on the detuning is observed (Fig. S7b), illustrating that multi-mode coupling over multiple optical octaves crucially relies on significant light-matter coupling strengths.

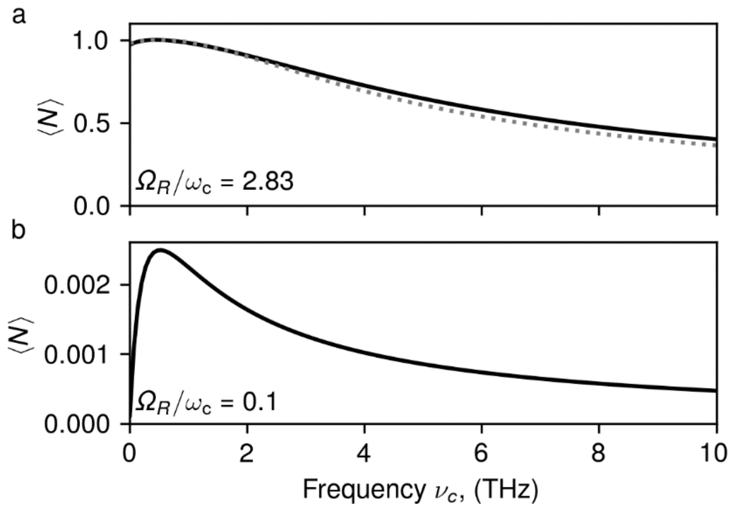

Figure S7: Virtual photon population $\langle N \rangle$ as a function of detuning with the cyclotron frequency $\boldsymbol{\nu_c}$ | a, A situation comparable to that of the structure featuring 48 QWs (black) with an equivalent single-mode coupling strength of $\Omega_{R,1}/\omega_1 = 2.83$, whereby only a single cavity and matter mode (grey dashed line) are considered with equivalent coupling strengths. **b**, For the single modes with a coupling strength of $\Omega_{R,1}/\omega_1 = 0.1$.

4. Subcycle time-domain quantum model

In order to compare the spectrum of coupled modes of our Hamiltonian to the spectra of the experimental data as well as the FEFD simulation, we derive the equations of motion for each operator using Heisenberg's equation of motion according to

$$\frac{d\hat{A}(t)}{dt} = -\frac{i}{\hbar}\left[\hat{A}(t), \hat{H}\right], \tag{14}$$

where $\hat{A}(t)$ is a bosonic operator representing the cavity or polariton modes. All couplings between modes result from expanding the commutator. Applying corresponding bosonic commutation relations, we derive a system of time-dependent differential equations which we subsequently treat by a mean-field approach, allowing us to introduce phenomenological dephasing rates and to include the driving field. For example, the dynamics of the cavity modes evolves according to

$$\dot{\alpha}_j = \langle \dot{\hat{a}}_j \rangle = -\frac{i}{\hbar}\langle \left[\hat{a}_j, \hat{H}\right] \rangle - \gamma_j \alpha_j + \mathcal{E}_{\mathrm{THz}}(t),$$

where $\alpha_j = \langle \hat{a}_j \rangle$ is the mean-field expectation value of the cavity electric field, γ_j is the damping rate, and $\mathcal{E}_{\mathrm{THz}}(t)$ is the THz far field which drives the cavity. We model the complex frequency response of the metasurface by a superposition of cavity fields α_j, each of which has a custom frequency ν_j, relative amplitude contribution, phase and damping rate such that their response optimally represents the far-field response obtained from FEFD calculations. Implementing a total of five oscillators with the parameters given in table S1, the FEFD result is faithfully reproduced (Fig. S8).

Our time-domain quantum model allows for investigating the role of each optical mode for the transmission spectrum of the structure, by a switch-off analysis. More specifically, the contributions of the most important cavity modes $j = 1$ and $j = 2$ to the far-field, are separately displayed in Fig. S10.

Mode index j	Frequency ν_j (THz)	Damping rate γ_j (THz)	Relative amplitude A_j	Phase ϕ_j
**1**	0.52	0.08	2.8	0
**2**	1.95	0.80	44.5	-0.14 π
**3**	3.75	0.12	0.8	-0.25 π
**4**	4.60	0.30	0.9	-0.6 π
**5**	6.00	0.30	7	-0.14 π

Table S1 | Parameters for the cavity modes used to model the frequency response of the resonator structure.

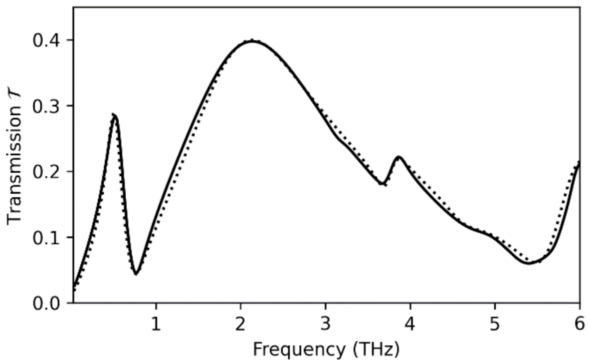

Figure S8 | Far-field transmission of the bare metasurface calculated by the FEFD method (solid curve) and transmission obtained from the harmonic oscillator representation of our time domain simulations (dotted curve).

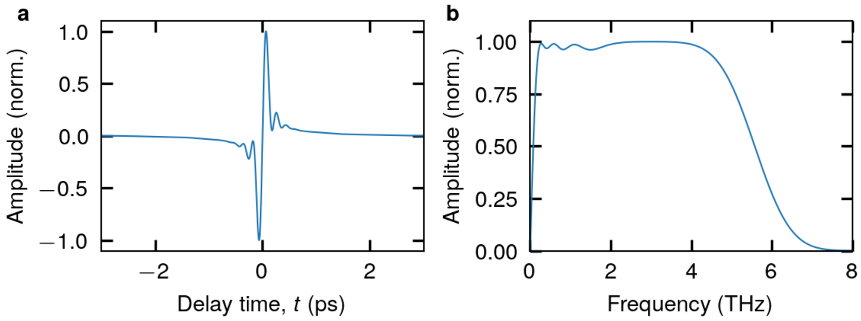

Figure S9 | Characteristics of THz excitation used in the time-domain theory. a, Waveform and **b**, amplitude spectrum.

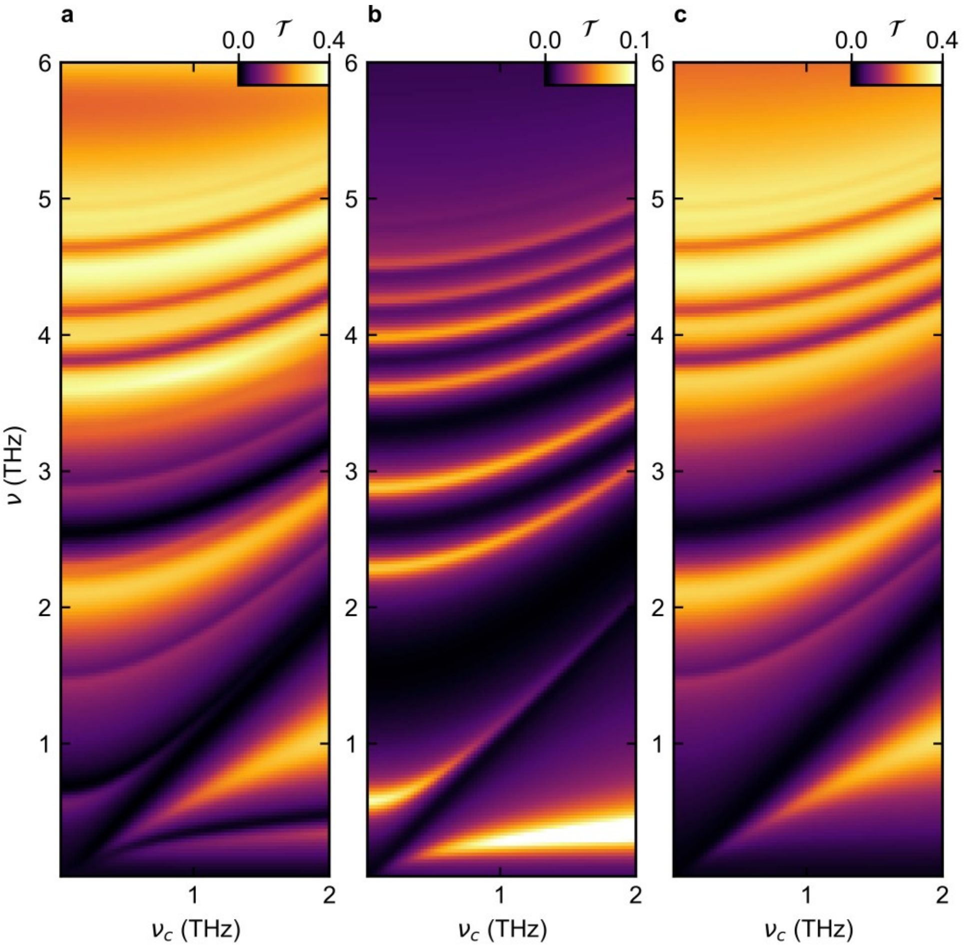

Figure S10 | Switch-off analysis for the optical modes. The time-domain simulation of the 48-QW structure includes **a,** all modes, **b**, only the first and **c**, only the second optical mode of the resonator structure.

As discussed in the main manuscript, our model allows us to access the microscopic polarization dynamics of the internal degrees of freedom of our multi-mode coupled structure. Owing to the very large coupling strengths $\Omega_{\mathrm{R},j=1,\alpha}$, all individual MP modes significantly influence each other by coupling to the same cavity mode. This effect is illustrated in the amplitude spectra in Fig. S11. While the spectral weight of each MP mode is centered near its intrinsic resonance frequency, they all share a common set of frequencies where local maxima are observed – a characteristic signature of very strong mutual coupling of oscillators.

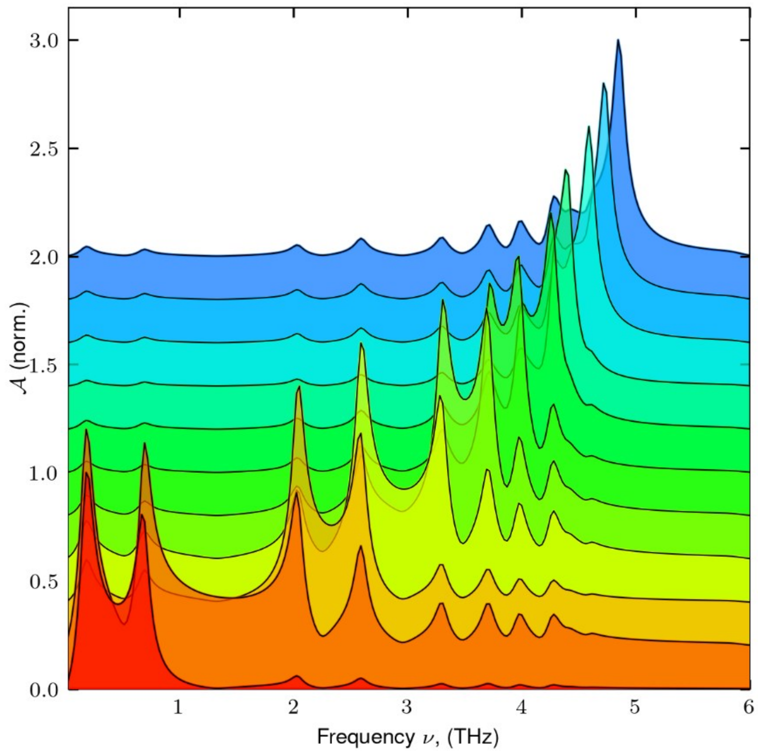

Figure S11 | Calculated spectra of the expectation values of the polarization of the MP modes $0 \leq \alpha \leq 10$. The spectra are each vertically offset by a value of 0.2, for clarity.

5. Parameter-free FEFD model

In addition to our time-domain quantum model, we calculate the optical response of the bare resonator structure by numerical FEFD simulations following the concept of Ref. [6]. The calculation yields the response of the coupled structures including the spatially resolved near-field distribution as well as the far-field transmission, without any free fit parameters. Our formalism requires the three-dimensional geometry of the nanostructure including the generally anisotropic dielectric response, implemented as a tensor function $\underline{\epsilon}(\vec{r}, \omega_{\mathrm{c}}, \omega)$ which depends on the position $\vec{r}$, the cyclotron frequency, ω_{c}, and the frequency of the light field, ω. Within the two-dimensional electron gas, $\underline{\epsilon}$ describes the gyrotropic nature of the cyclotron resonance in x and y direction owing to the z-polarized static magnetic bias field. In the z direction, we employ the background dielectric constant, since the small thickness of the quantum wells leads to a plasma frequency far above the frequencies of interest for our structure. Additionally, we reduce the numerical complexity by modelling the quantum well stack including its

barriers by an effective-medium approach with an effective dielectric tensor. The magnetically invariant background and substrate layers are implemented by the dielectric function $\epsilon_{\mathrm{GaAs}}(\omega) = \epsilon_\infty \frac{\omega_{\mathrm{LO}}^2-\omega^2+i\gamma_{\mathrm{LO}}\omega}{\omega_{\mathrm{TO}}^2-\omega^2+i\gamma_{\mathrm{TO}}\omega}$, including the optical phonons of GaAs to improve the accuracy of the calculation especially at higher THz frequencies. Here we use $\epsilon_\infty = 10.87$ [10], $\omega_{\mathrm{LO}}/2\pi = 8.839$ THz, $\omega_{\mathrm{TO}}/2\pi = 8.124$ THz, $\gamma_{\mathrm{LO}} = 0.0225$ THz and $\gamma_{\mathrm{TO}} = 0.0255$ THz [11]. A single unit cell of the metasurface is implemented and periodically extended in x and y direction by periodic boundary conditions. The resulting far-field calculations predict experimental results across the entire spectral range with high accuracy (Fig. S12 and S13).

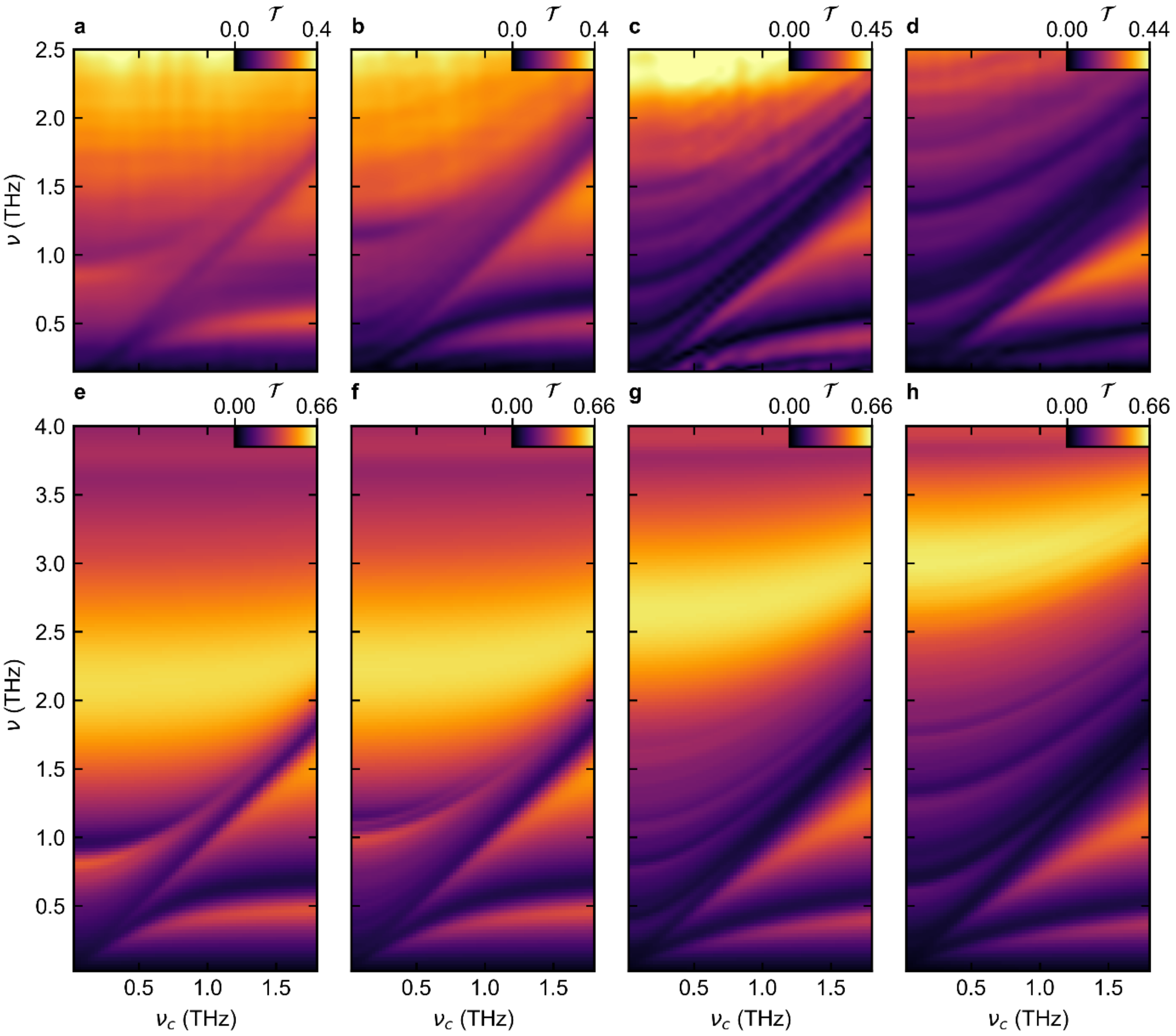

Figure S12 | Comparison of experimental spectra and FEFD calculations. a, Experimental THz magneto-transmission as a function of ν_c for the single-QW, **b,** 3-QW, **c,** 6-QW, and **d,** 12-QW structure. **e-h** Corresponding FEFD THz transmission data.

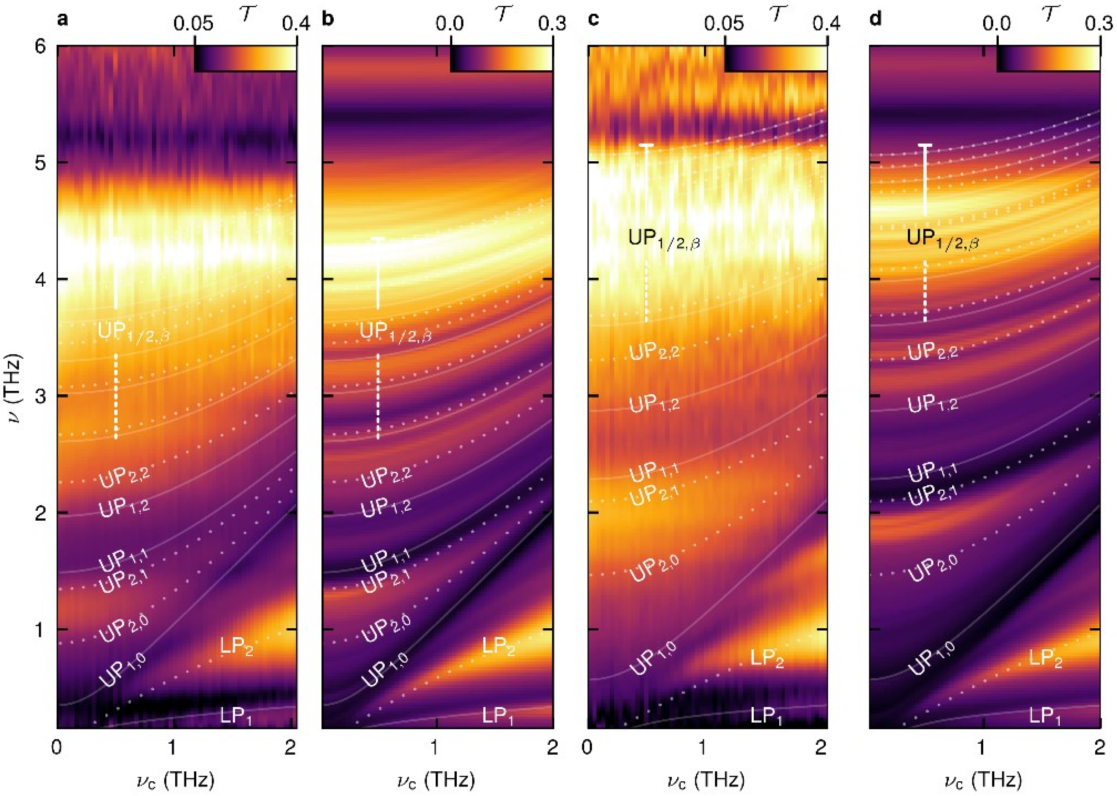

Figure S13 a, THz magneto-transmission of the 24-QW sample as a function of ν_c (see Fig. 3). Calculated polariton frequencies (solid & dashed curves) with distinct resonances marked. **b**, FEFD simulated transmission and polariton frequencies. **c**, Transmission of the 48-QW structure. **d**, FEFD simulated transmission and identical polariton frequencies.

6. Scaling of the coupling strength

Following Hagenmüller et al. [12], the coupling strength of an ensemble of electronic oscillators coupled to a cavity mode scales with the square root of the total carrier density in the QW stack, $\Omega_R \propto \rho^{0.5}$. This dependency is perfectly reproduced for our first four structures containing 1, 3, 6 or 12 QWs. However, owing to limited penetration depth of the near-field into the QW stacks, we observe a deviation from this ideal scaling law for the structures with 24 and 48 QWs (Fig. S14). Nevertheless, the coupling strength still increases significantly even including this effect.

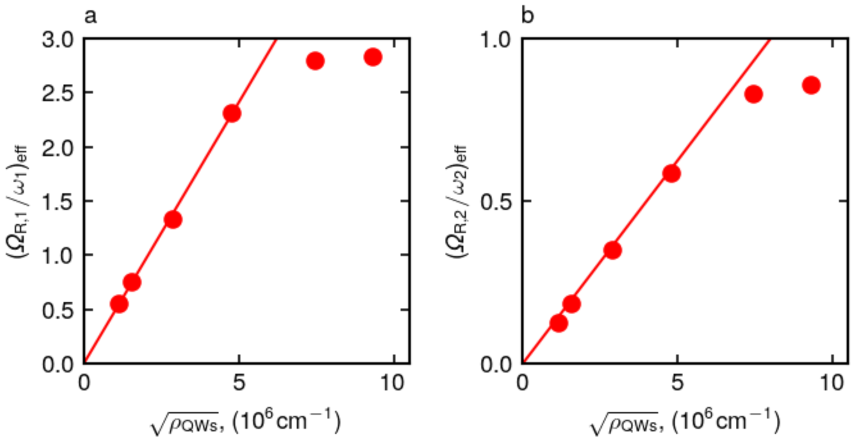

Figure S14 | Scaling of the coupling strength with the number of electronic oscillators. a, Equivalent coupling strength $\Omega_{R,1}/\omega_1$ for the first cavity mode as a function of the square root of the total charge carrier density, and linear fit of the data adjusted to the first four data points. **b**, Equivalent data for the second cavity mode.

7. Additional data visualization

Figs. S15 and S16 show the data of the colour plots of the main manuscript as waterfall plots as an alternative visualization of the frequency tuning of the light-matter coupled modes as a function of the cyclotron frequency. The curves are vertically offset for clarity.

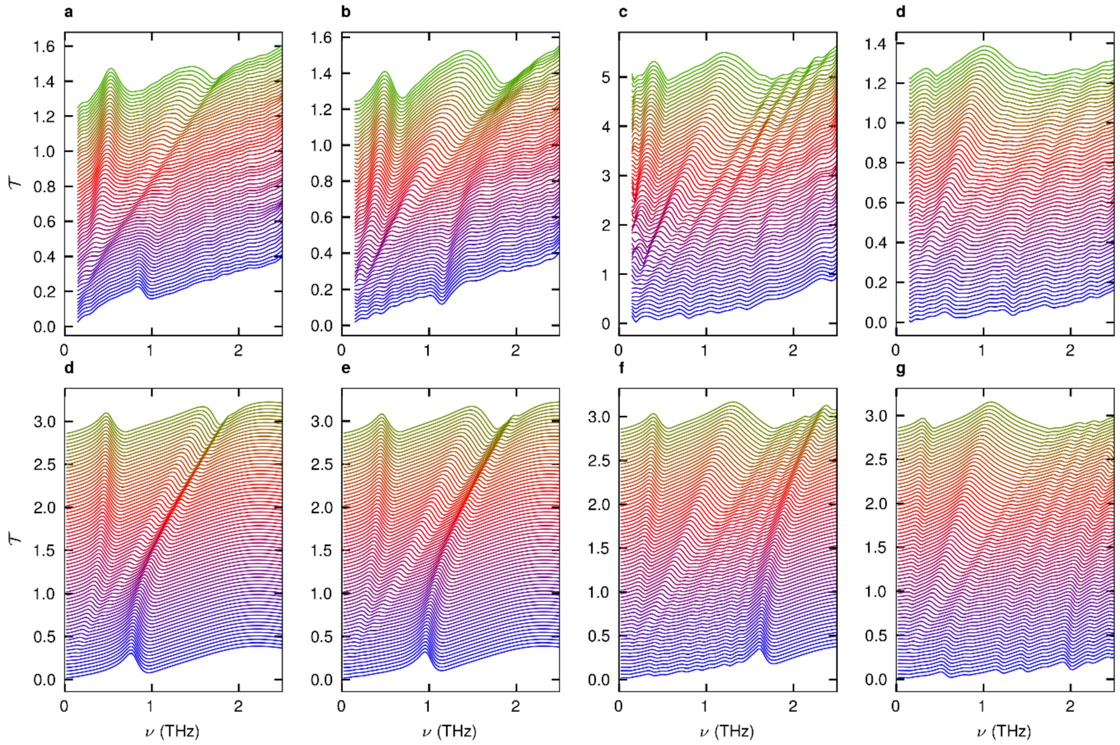

Figure S15 | Waterfall plots of Figure 2. a, THz magneto-transmission as a function of ν_c of the single-QW structure. **d**, Spectrum obtained from time-domain quantum model **b**, Transmission of the 3-QW structure and **e**, simulation. **c** Transmission of the 6-QW structure, and **f**, simulation. **d**, Transmission of the 12-QW structure, and **g**, simulation.

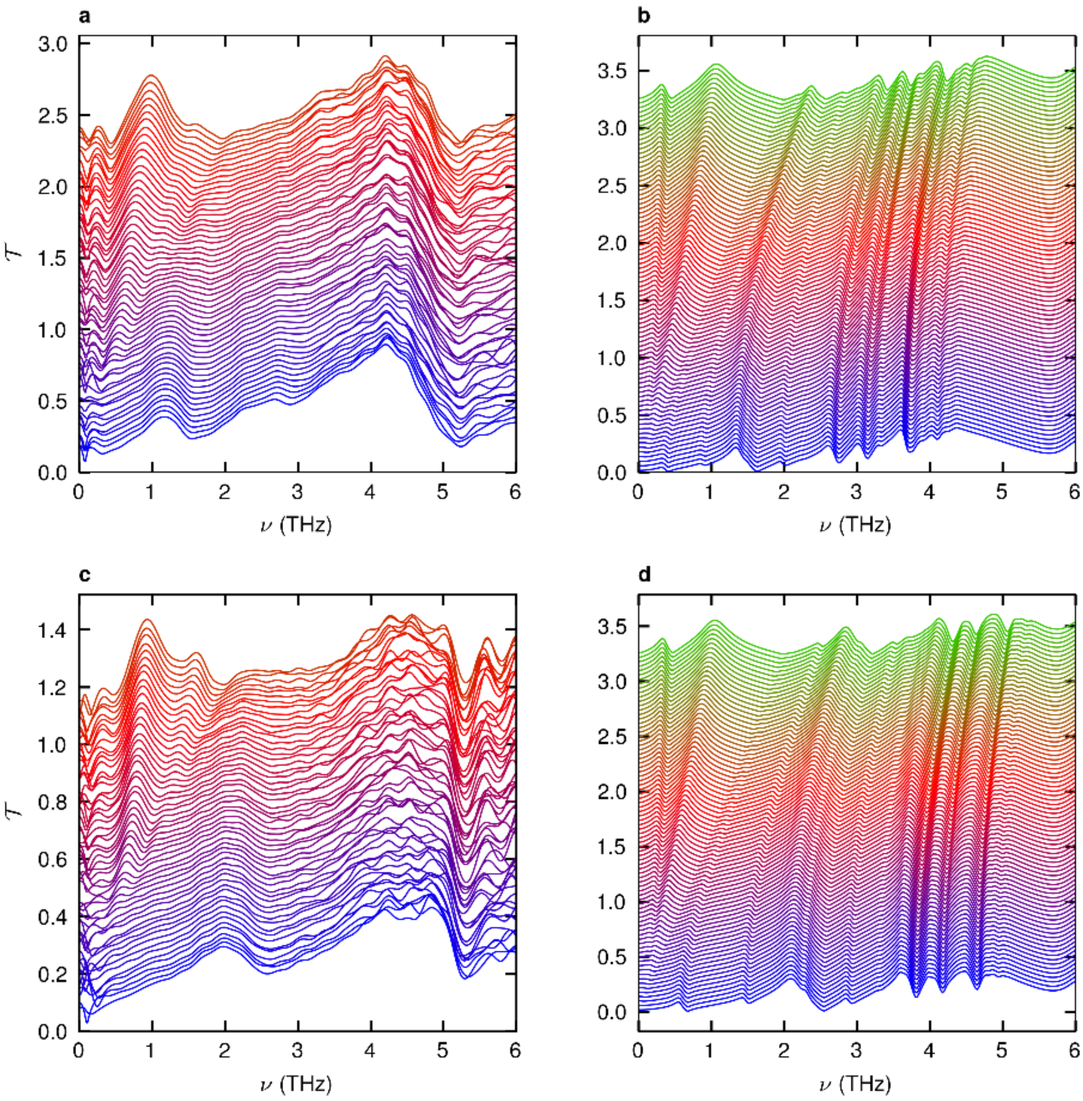

Figure S16 | Waterfall plots of the data of Fig. 3. **a**, THz magneto-transmission of the 24-QW sample as a function of ν_c. **b**, Corresponding calculated transmission. **c**, Transmission of the 48-QW structure. **d**, Corresponding calculated transmission.

References:

[1] J. Keller *et al.*, Advanced Optical Materials **5**, 1600884 (2017).

[2] F. Stern, Phys. Rev. Lett. **18**, 546 (1967).

[3] V. V. Popov, O. V. Polischuk, and M. S. Shur, Journal of Applied Physics **98**, 33510 (2005).

[4] M. S. Kushwaha, Surface Science Reports **41**, 1 (2001).

[5] A. Eguiluz, T. K. Lee, J. J. Quinn, and K. W. Chiu, Phys. Rev. B **11**, 4989 (1975).

[6] A. Bayer, M. Pozimski, S. Schambeck, D. Schuh, R. Huber, D. Bougeard, and C. Lange, Nano Lett. **17**, 6340 (2017).

[7] I. V. Bondarev and V. M. Shalaev, Opt. Mater. Express, OME **7**, 3731 (2017).

[8] Backes, Peeters, Brosens, and Devreese, Physical review. B, Condensed matter **45**, 8437 (1992).

[9] C. Ciuti, G. Bastard, and I. Carusotto, Phys. Rev. B **72** (2005).

[10] D. J. Lockwood, G. Yu, and N. L. Rowell, Solid State Communications **136**, 404 (2005).

[11] G. Irmer, M. Wenzel, and J. Monecke, phys. stat. sol. (b) **195**, 85 (1996).

[12] D. Hagenmüller, S. de Liberato, and C. Ciuti, Phys. Rev. B **81** (2010).
